# Detoxifying Effects of a Phenolic-Rich *Thunbergia laurifolia* Water Extract Against Chlorpyrifos-Induced Oxidative Stress: Evidence from an Integrated In Vitro and In Vivo Study

**DOI:** 10.3390/ijms27177876

**Published:** 2026-09-03

**Authors:** Phraepakaporn Kunnaja, Supaporn Intatham, Parirat Khonsung, Kanjana Jaijoy, Thunyatorn Yimsoo, Piyanuch Rojsanga, Seewaboon Sireeratawong

**Affiliations:** 1 Department of Medical Technology, Faculty of Associated Medical Sciences, Chiang Mai University, Chiang Mai 50200, Thailand; phraepakaporn.k@cmu.ac.th; 2 Department of Pharmacology, Faculty of Medicine, Chiang Mai University, Chiang Mai 50200, Thailand; intatham_s@outlook.com (S.I.); parirat.khons@cmu.ac.th (P.K.); 3 Clinical Research Center for Food and Herbal Product Trials and Development (CR-FAH), Faculty of Medicine, Chiang Mai University, Chiang Mai 50200, Thailand; 4 McCormick Faculty of Nursing, Payap University, Chiang Mai 50000, Thailand; kanjana_j@payap.ac.th; 5 Laboratory Animal Center, Office of Advanced Science and Technology, Thammasat University, Pathum Thani 12120, Thailand; tyimsoo@tu.ac.th; 6 Department of Pharmaceutical Chemistry, Faculty of Pharmacy, Mahidol University, Bangkok 10400, Thailand; piyanuch.roj@mahidol.ac.th

**Keywords:** *Thunbergia laurifolia*, caffeic acid, rosmarinic acid, detoxification, oxidative stress, apoptosis, chlorpyrifos, acetylcholinesterase

## Abstract

*Thunbergia laurifolia* has long been used in traditional medicine for detoxification; however, the biological basis of its detoxifying effects remains poorly understood. This study investigated the detoxifying effects of a phenolic-rich aqueous extract of *T. laurifolia* using integrated in vitro and in vivo approaches. Caffeic acid (CA) and rosmarinic acid (RA) were used as marker compounds for extract standardization. The aqueous extract was evaluated for antioxidant, acetylcholinesterase, and apoptosis-modulating activities in vitro, followed by evaluation in a chlorpyrifos-induced oxidative stress model in vivo. The extract exhibited antioxidant activity and maintained acetylcholinesterase (AChE) activity. In LX-2 hepatic stellate cells, the extract induced predominantly early apoptosis, whereas CA and RA induced predominantly late apoptosis. In chlorpyrifos-exposed rats, the extract restored AChE activity, reduced lipid peroxidation, and enhanced endogenous antioxidant defenses, as evidenced by decreased malondialdehyde (MDA) levels and increased glutathione (GSH) levels and superoxide dismutase (SOD) activity. Collectively, these findings suggest that attenuation of oxidative stress may represent one of the mechanisms underlying the detoxifying effects traditionally attributed to *T. laurifolia*.

## 1. Introduction

*Thunbergia laurifolia* Lindl. is a medicinal plant widely distributed in tropical regions, including Southeast Asia, where it has been traditionally used as an antidote for various toxic substances, particularly pesticides, heavy metals, and drugs. In Thai traditional medicine, decoctions prepared from its leaves have also been used for reducing fever and alleviating symptoms associated with toxic exposure [1]. Despite its long-standing ethnopharmacological use, the underlying biological mechanisms responsible for its detoxifying effects remain incompletely understood. Accumulating evidence suggests that oxidative stress plays a central role in the pathogenesis of toxin-induced cellular damage and organ dysfunction. Excessive production of reactive oxygen species (ROS) leads to lipid peroxidation, mitochondrial dysfunction, and dysregulation of cell death pathways, ultimately contributing to tissue injury and fibrosis. Antioxidant defense systems, including enzymatic components such as superoxide dismutase (SOD) and glutathione (GSH), are therefore critical in maintaining cellular redox homeostasis and protecting against oxidative damage.

Previous pharmacological studies have demonstrated a broad range of biological activities of *T. laurifolia*, including antioxidant, anti-inflammatory, antipyretic, antidiabetic, neuroprotective, gastroprotective, and antidotal effects [2,3,4,5,6,7,8,9,10,11,12]. Furthermore, *T. laurifolia* has long been used in traditional medicine for detoxification in cases of poisoning, and several experimental studies have supported its protective effects against toxic substance-induced oxidative injury [2,13]. Consistent with its traditional use, aqueous extracts of *T. laurifolia* have also been reported to attenuate alcohol-induced toxicity by reducing blood alcohol levels and enhancing alcohol dehydrogenase and aldehyde dehydrogenase activities. In addition, the extracts decreased hepatic lipid peroxidation, thereby contributing to hepatocellular protection against oxidative damage [10,14]. The mechanisms underlying the detoxifying activity of *T. laurifolia*, particularly in relation to oxidative stress regulation and apoptosis modulation, remain poorly understood. In particular, how the antioxidant and apoptosis-modulating activities of *T. laurifolia* coordinately contribute to its detoxifying effects during toxicant-induced liver injury remains unclear. Effective detoxification likely involves coordinated mechanisms that preserve normal hepatocytes from oxidative injury while facilitating the removal of damaged or activated cells. Activated hepatic stellate cells are highly responsive to oxidative stress and play important roles in hepatic injury and tissue remodeling. Therefore, LX-2 human hepatic stellate cells were used as a relevant in vitro model to evaluate whether *T. laurifolia* modulates apoptosis-related cellular responses under conditions associated with toxicant-induced liver injury [15,16].

The biological activities of *T. laurifolia* have been associated with several phytochemicals, including caffeic acid (CA) and rosmarinic acid (RA), which possess potent antioxidant and redox-modulating properties. More recently, detoxifying formulations containing *T. laurifolia* have demonstrated detoxifying effects against chlorpyrifos-induced toxicity in experimental models. Chlorpyrifos (CPF) was selected as the experimental model because it is one of the most widely used organophosphate pesticides worldwide and a common source of pesticide-related toxicity [17]. In addition to its well-established inhibition of acetylcholinesterase (AChE), CPF induces oxidative stress and cellular injury in multiple organs [18,19]. Since *T. laurifolia* has traditionally been used as a detoxifying herb for individuals exposed to agricultural chemicals and pesticides [1,20], CPF-induced toxicity provides a relevant experimental model for evaluating the biological basis of its traditional detoxifying effects. In the present study, the term “detoxification” refers to the attenuation of toxic effects and the enhancement of endogenous defense mechanisms, particularly through the reduction in oxidative stress, preservation of antioxidant defenses, and maintenance of physiological homeostasis following toxicant exposure [17,19]. A formulation containing *T. laurifolia* and *Embelia sessiliflora* was shown to attenuate oxidative stress, restore AChE activity, reduce lipid peroxidation, and preserve hepatic histoarchitecture in CPF-exposed rats. The formulation also exhibited strong antioxidant and pro-apoptotic activities in hepatic stellate cells, suggesting that modulation of oxidative stress and apoptosis may contribute to its detoxifying effects [20].

Despite the numerous reports describing antioxidant, anti-inflammatory, hepatoprotective, and other pharmacological activities of *T. laurifolia*, substantial differences remain among studies with respect to extraction methods, experimental models, and biological endpoints. Most investigations have focused on general pharmacological effects, whereas relatively little attention has been given to the biological processes that may underlie its traditional use as a detoxifying herb. In particular, the relationships among antioxidant defense mechanisms, AChE activity, and apoptosis-related responses in pesticide-induced toxicity remain incompletely understood. Therefore, the studies integrating both in vitro and in vivo approaches are needed to provide biological evidence supporting its traditional detoxifying use.

Accordingly, we hypothesized that *T. laurifolia* exerts its traditional detoxifying effects through attenuation of oxidative stress and modulation of cellular responses to toxicant exposure, thereby contributing to the maintenance of cellular homeostasis. To test this hypothesis, the present study integrated phytochemical characterization with complementary in vitro and in vivo evaluations of *T. laurifolia.* The in vitro investigations assessed antioxidant activity, apoptosis-modulating effects in LX-2 human hepatic stellate cells as a model of cellular responses to oxidative stress, and modulation of AChE activity. The in vivo detoxifying potential was subsequently evaluated in a CPF-induced oxidative stress model to determine whether these biological properties contributed into attenuation of CPF-induced biochemical dysfunction.

## 2. Results

### 2.1. Phytochemical Characterization and Standardization of T. laurifolia Leaf Extract

#### 2.1.1. Extraction Yield

The aqueous extract of *T. laurifolia* leaves yielded 52.8% (*w*/*w*) dried extract, corresponding to an extractive ratio of 1:1.9. Chemical standardization demonstrated the presence of caffeic acid (CA) and rosmarinic acid (RA), which were quantified as marker compounds in the extract. The extraction yield, extractive ratio, and contents of CA and RA are presented in Table 1.

**Table 1:** Yield, extractive ratio, and contents of caffeic acid and rosmarinic acid in *T. laurifolia* leaf extract.

Yield (%*w*/*w*)	Extractive Ratio	CA (mg/g Extract)	RA (mg/g Extract)
52.8	1:1.9	0.53 ± 0.00	2.14 ± 0.01

Values are expressed as mean ± SD (n = 3).

#### 2.1.2. TLC Fingerprint

**Figure 1:**
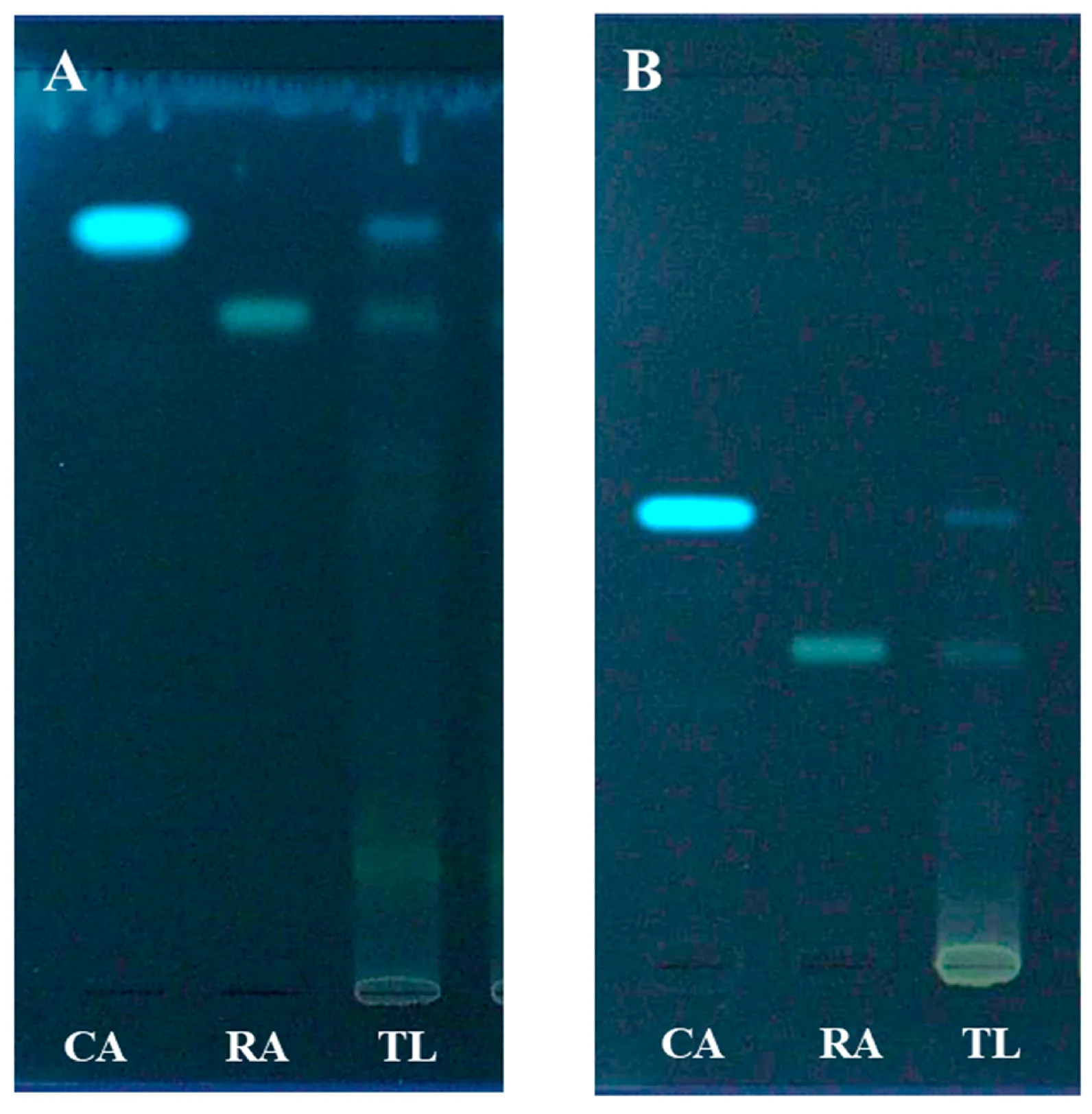
TLC chromatograms of *T. laurifolia* leaf extract after derivatization with NP/PEG reagent and visualization under UV light at 366 nm. Silica gel 60 F254 was used as the stationary phase. (**A**) Solvent system I: chloroform:methanol:formic acid (70:30:5). (**B**) Solvent system II: toluene:ethyl acetate:formic acid (10:9:2). CA = caffeic acid; RA = rosmarinic acid; TL = *T. laurifolia* extract.

Thin-layer chromatography (TLC) analysis confirmed the presence of these phenolic compounds. The extract exhibited spots corresponding to CA and RA, as identified by comparison with reference standards based on hRf values in two solvent systems. Caffeic acid showed hRf values of 85 and 50, while rosmarinic acid showed hRf values of 71 and 37 in solvent systems I and II, respectively (Figure 1).

#### 2.1.3. HPLC Fingerprint of Major Phenolic Compounds

**Figure 2:**
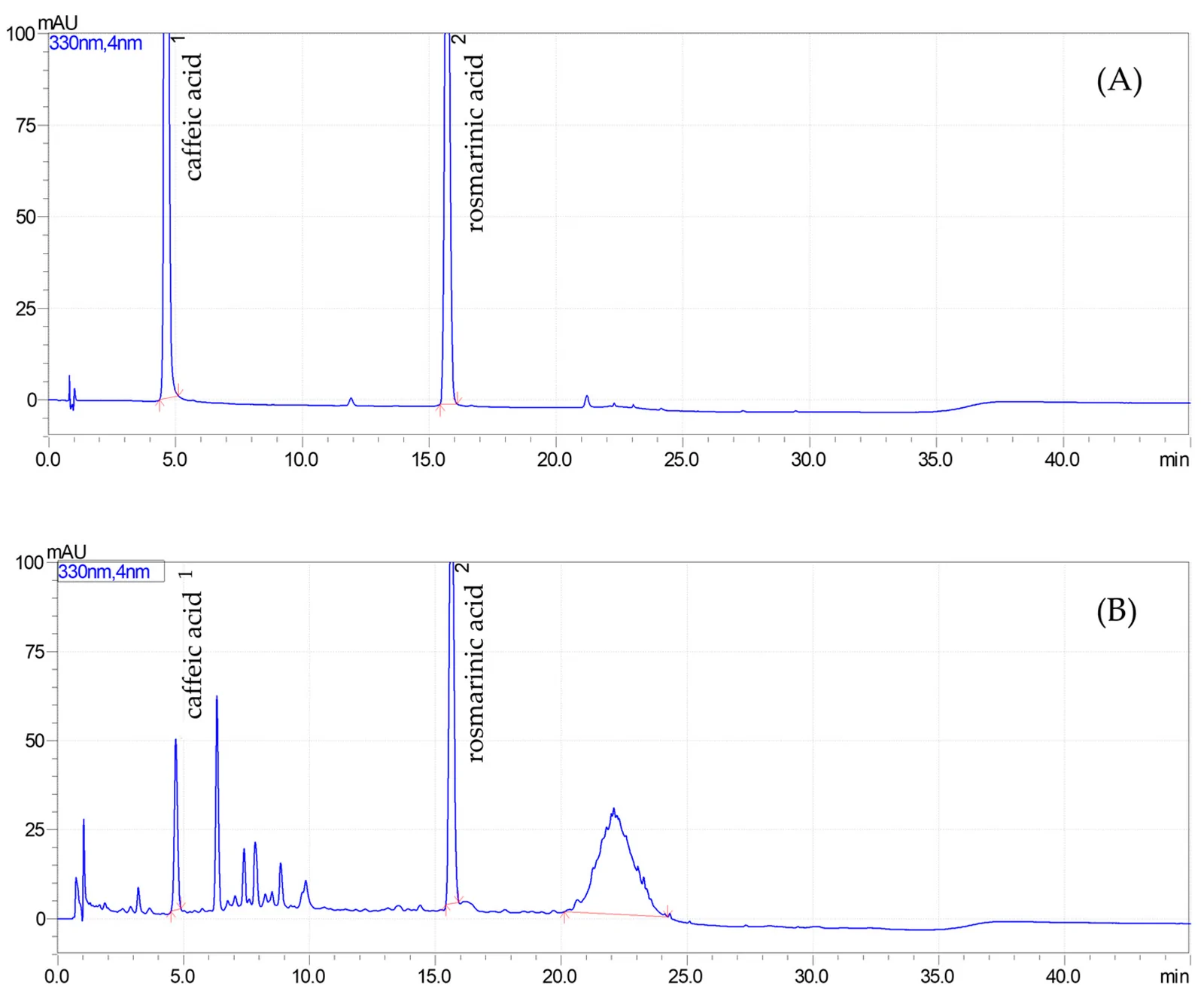
HPLC chromatograms of (**A**) standard compounds, caffeic acid and rosmarinic acid (50 µg/mL), and (**B**) aqueous extract of *Thunbergia laurifolia* leaves (2 mg/mL), detected at 330 nm. Separation was performed using an Agilent^®^ SB-C18 column (3.0 × 150 mm, 5 µm). The mobile phase consisted of 0.02% phosphoric acid in water and acetonitrile. Peaks 1 and 2 correspond to caffeic acid and rosmarinic acid, respectively.

High-performance liquid chromatography (HPLC) further verified the presence of these compounds. The chromatographic profile of the extract showed peaks corresponding to CA and RA, consistent with those of the standard compounds at 330 nm (Figure 2). These results confirmed the presence of CA and RA in the aqueous extract and supported their use as phytochemical markers for extract characterization.

### 2.2. In Vitro Antioxidant Activity

The antioxidant activities of *T. laurifolia* extract were evaluated using DPPH, superoxide radical scavenging, ABTS, and FRAP assays (Table 2). The extract exhibited antioxidant activity in all assays, demonstrating its ability to scavenge free radicals and reduce ferric ions. Among the radical scavenging assays, the extract showed the lowest IC_50_ value in the DPPH assay, followed by the superoxide radical scavenging assay. In addition, antioxidant capacity was demonstrated in both the ABTS and FRAP assays. The isolated phenolic compounds, CA and RA, exhibited lower IC_50_ values in the DPPH and superoxide radical scavenging assays, as well as greater antioxidant capacities in the ABTS and FRAP assays, than the crude extract.

### 2.3. In Vitro Acetylcholinesterase (AChE) Activity

**Figure 3:**
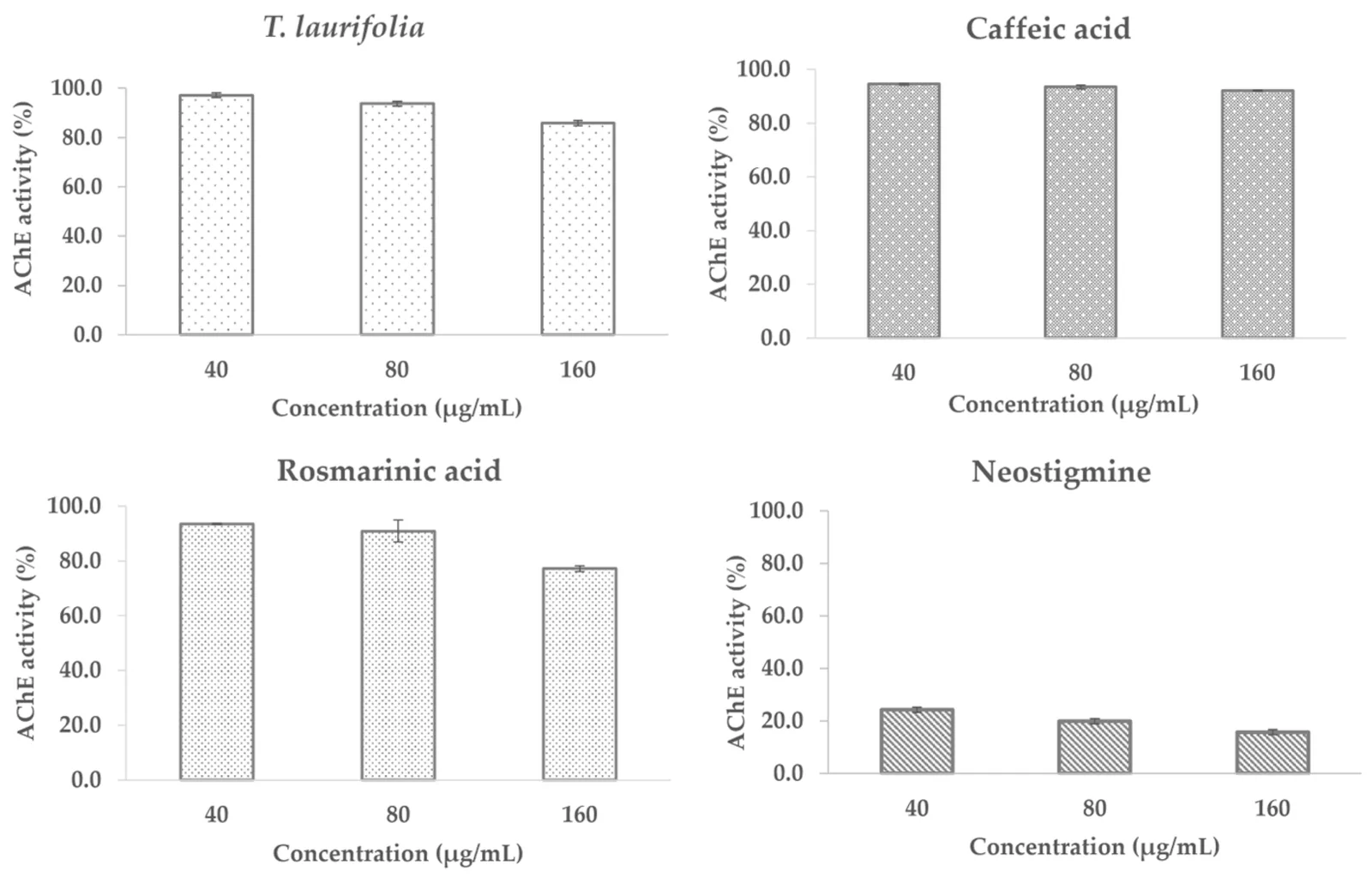
Effects of *T. laurifolia* extract, caffeic acid, rosmarinic acid, and neostigmine on AChE activity. AChE activity was expressed as a percentage relative to the untreated control (100%). *T. laurifolia* extract and its major phenolic constituents maintained relatively high AChE activity across the tested concentration range, whereas neostigmine markedly reduced enzyme activity. Data are presented as mean ± SEM (n = 3).

AChE activities of *T. laurifolia* extract, CA, RA, and neostigmine are presented in Figure 3. *T. laurifolia* extract had minimal effects on AChE activity, with enzyme activity remaining above 85% across all tested concentrations. CA and RA similarly maintained relatively high AChE activity, although RA showed a greater effect at the highest concentration tested. In contrast, neostigmine markedly reduced AChE activity.

### 2.4. Effects of T. laurifolia Extract on LX-2 Hepatic Stellate Cells

#### 2.4.1. Effects on LX-2 Cell Proliferation

**Figure 4:**
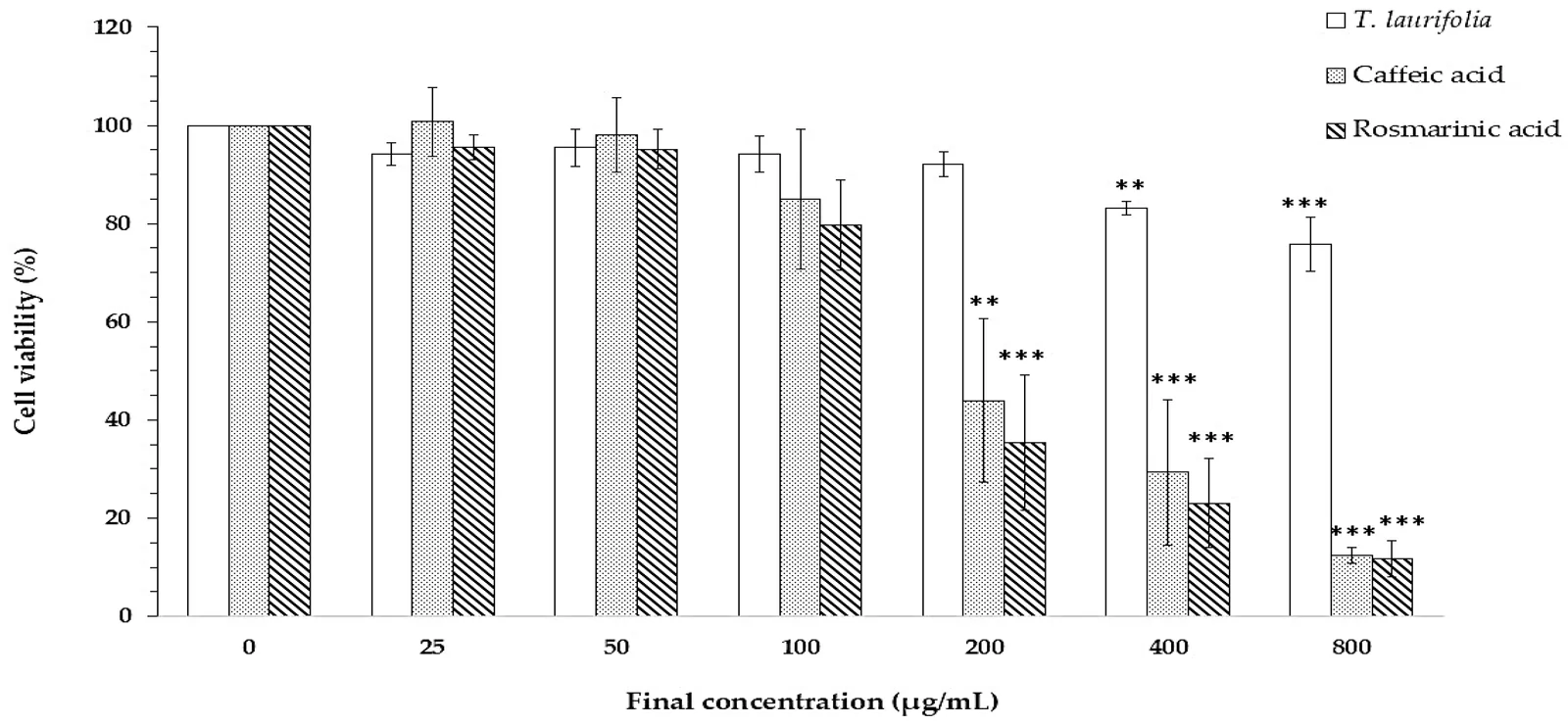
Effects of *T. laurifolia* extract and the purified compounds caffeic acid (CA) and rosmarinic acid (RA) on LX-2 cell viability after 24 h of treatment. Data are presented as the mean ± SEM of three independent experiments. ** *p* < 0.01 and *** *p* < 0.001 versus the untreated control cells (0 µg/mL).

After 24 h of incubation, treatment with *T. laurifolia* extract and the purified compounds CA and RA significantly reduced LX-2 cell viability compared with the untreated control cells. Significant cytotoxic effects were observed at extract concentrations of 400–800 µg/mL and at CA and RA concentrations of 200–800 µg/mL. Cell death occurred in a concentration-dependent manner, as shown in Figure 4.

#### 2.4.2. Effects on Apoptosis in LX-2 Cells

**Figure 5:**
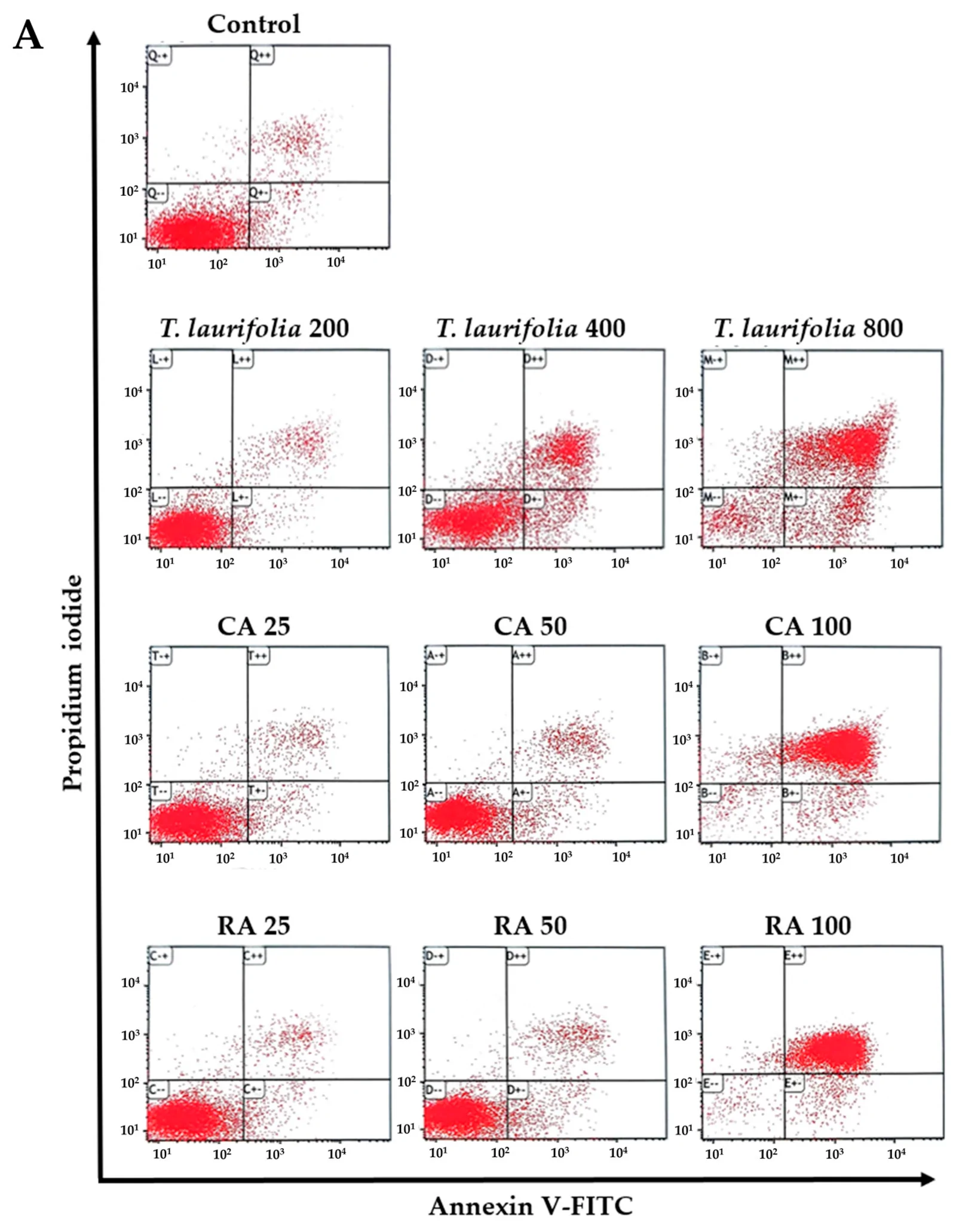

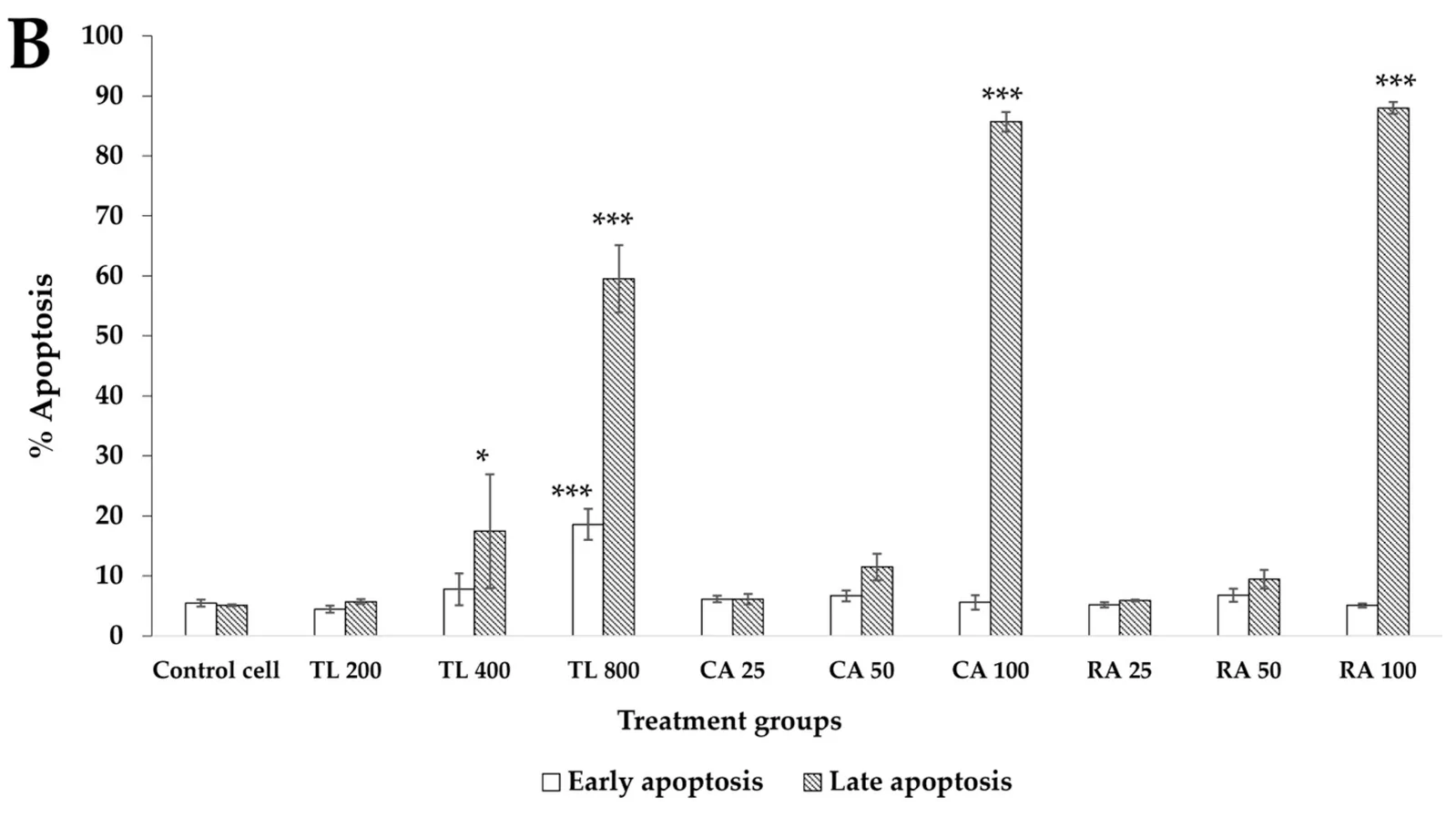
Effects of *T. laurifolia* extract (TL), caffeic acid (CA), and rosmarinic acid (RA) on apoptosis in LX-2 human hepatic stellate cells. (**A**) Representative Annexin V-FITC/PI flow cytometric dot plots after treatment with *T. laurifolia* extract (200–800 μg/mL), CA (25–100 μg/mL), and RA (25–100 μg/mL). Lower left quadrant (Annexin V−/PI−) represents viable cells; lower right quadrant (Annexin V+/PI−) represents early apoptotic cells; upper right quadrant (Annexin V+/PI+) represents late apoptotic cells; and upper left quadrant (Annexin V−/PI+) represents necrotic cells. (**B**) Quantitative analysis of apoptotic cells. Data are presented as mean ± SEM (n = 3). * *p* < 0.05 and *** *p* < 0.001 vs. control.

Based on the cell proliferation assay, concentrations showing biological activity in LX-2 cells were selected for apoptosis analysis. Preliminary experiments using CA and RA at concentrations of 200–800 µg/mL resulted predominantly in late apoptotic cell death (>80%) with minimal induction of early apoptosis. Therefore, lower concentrations (25, 50, and 100 µg/mL) were selected for subsequent experiments. Treatment with CA and RA at 25 and 50 µg/mL did not significantly affect apoptotic cell populations in LX-2 cells. However, both compounds at 100 µg/mL significantly increased the proportion of late apoptotic cells compared with the untreated control group (*p* < 0.001), whereas no significant changes in early apoptosis were observed. In contrast, the crude *T. laurifolia* extract exhibited a distinct apoptotic profile. Treatment with 400 µg/mL significantly increased late apoptosis (*p* < 0.05). Treatment with 800 µg/mL significantly increased early apoptotic cells while also increasing late apoptosis. (*p* < 0.001). Representative flow cytometric dot plots and quantitative analysis of apoptotic cells are shown in Figure 5A and Figure 5B, respectively.

### 2.5. Anti-Pesticide Activity

#### 2.5.1. Effects on Body Weight

Rats in all treatment groups received CPF at a dose of 16 mg/kg body weight daily for 16 days. The CPF-treated rats exhibited a trend toward reduced body weight gain compared with the normal control group; however, this difference did not reach statistical significance. Likewise, treatment with *T. laurifolia* extract did not significantly alter body weight relative to the CPF control group throughout the experimental period (Table 3).

#### 2.5.2. Effects on Organ Weight

After 16 days of treatment, liver and kidney tissues were collected for evaluation. No significant differences in liver or kidney weights were observed between the normal control and CPF control groups (Table 4). Likewise, treatment with *T. laurifolia* extract did not significantly affect liver weight compared with the CPF control group. However, rats treated with the highest dose of *T. laurifolia* extract (2468 mg/kg) exhibited a slight but statistically significant increase in kidney weight relative to the CPF control group. The biological significance of this finding should be interpreted in conjunction with the histopathological and biochemical results.

#### 2.5.3. Histopathological Findings

**Figure 6:**
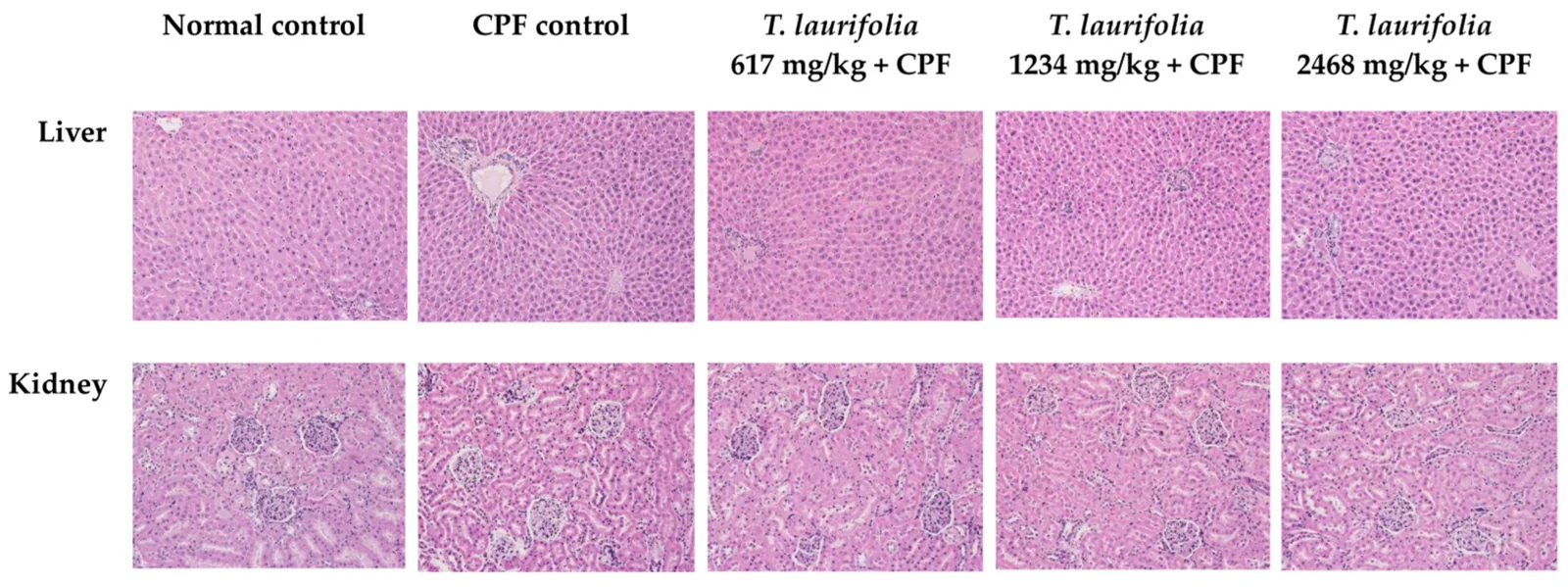
Representative hematoxylin and eosin (H&E)-stained sections of liver and kidney tissues from the normal control, CPF control, and *T. laurifolia* extract-treated groups (617, 1234, and 2468 mg/kg). No remarkable histopathological abnormalities were observed in the liver or kidney tissues of any experimental group. Representative photomicrographs were captured at 10× magnification.

Histopathological examination revealed no overt pathological alterations in the liver or kidney tissues of any experimental group, indicating that the CPF regimen used in this study did not induce detectable tissue injury under the conditions tested (Figure 6).

#### 2.5.4. Effects on Hematological Parameters

Hematological analysis revealed that chlorpyrifos exposure significantly increased red blood cell (RBC) count, hematocrit (Hct), and monocyte (Mono) count compared with the normal control group (*p* < 0.05). Administration of *T. laurifolia* extract ameliorated these CPF-induced hematological alterations, with RBC, hemoglobin (Hb), and Hct showing a trend toward the values observed in the normal control group. Mono counts were significantly reduced in all extract-treated groups compared with the CPF control group (*p* < 0.05), with values approaching those of the normal control group. No significant differences were observed in the remaining hematological parameters among the experimental groups (Table 5 and Table 6).

#### 2.5.5. Effects on Serum Chemical Parameters

Serum chemical analysis showed that chlorpyrifos exposure significantly increased serum creatinine (Cr) levels compared with the normal control group (*p* < 0.05), whereas most of the remaining serum chemical parameters were not significantly altered (Table 7). Administration of *T. laurifolia* extract at doses of 617 and 1234 mg/kg significantly reduced serum Cr levels compared with the CPF control group (*p* < 0.05), with values approaching those of the normal control group. Total protein (TP) levels were significantly lower in the 1234 mg/kg group than in the CPF control group (*p* < 0.05), although the absolute difference was small. Serum aspartate aminotransferase (AST) levels did not differ significantly among the experimental groups; however, rats treated with 1234 and 2468 mg/kg extract exhibited a trend toward lower values than the CPF control group. Likewise, serum alanine aminotransferase (ALT) levels in the 1234 and 2468 mg/kg groups were lower than those in the CPF control group, although the differences were not statistically significant. In contrast, the 617 mg/kg group showed a significantly higher serum ALT level than the CPF control group (*p* < 0.05).

#### 2.5.6. Effects on Oxidative Stress Biomarkers

The effects of *T. laurifolia* extract on oxidative stress biomarkers in chlorpyrifos-treated rats are summarized in Table 8. Chlorpyrifos exposure significantly increased serum malondialdehyde (MDA) levels, while significantly decreasing glutathione (GSH) levels and superoxide dismutase (SOD) activity compared with the normal control group, indicating enhanced oxidative stress. Pre-treatment with *T. laurifolia* extracts significantly attenuated these alterations across the tested doses. Treatment with the extract at doses of 617, 1234, and 2468 mg/kg significantly reduced serum MDA levels compared with the chlorpyrifos-treated group. In addition, the extract increased serum GSH levels and restored SOD activity, particularly at the higher doses, with values approaching those observed in the normal control group.

#### 2.5.7. Effects on Blood AChE Activity

**Figure 7:**
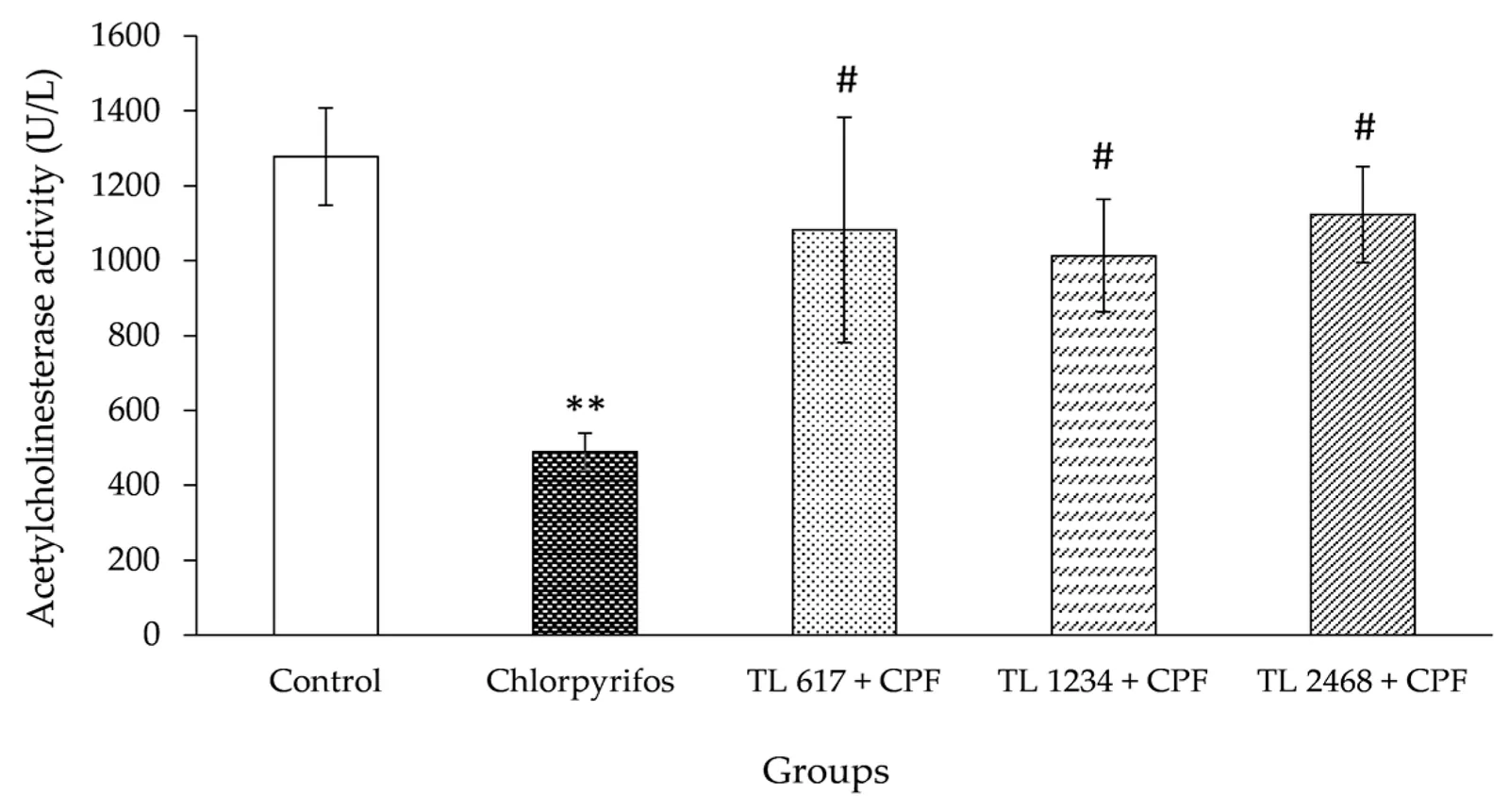
Effects of *T. laurifolia* extract on blood AChE activity in CPF-treated rats. CPF treatment significantly decreased blood AChE activity compared with the normal control group. Pre-treatment with *T. laurifolia* extracts at doses of 617, 1234, and 2468 mg/kg significantly increased AChE activity compared with the CPF-treated group. Data are expressed as mean ± SEM (n = 6). ** *p* < 0.01 versus the normal control group; # *p* < 0.05 versus the CPF-treated group.

Blood AChE activity is shown in Figure 7. Chlorpyrifos treatment significantly decreased AChE activity compared with the normal control group (*p* < 0.01). Pre-treatment with *T. laurifolia* extracts at doses of 617, 1234, and 2468 mg/kg significantly increased AChE activity compared with the chlorpyrifos-treated group (*p* < 0.05). The increase was observed at all tested doses, indicating a protective effect.

## 3. Discussion

*Thunbergia laurifolia* has long been used in Thai traditional medicine as a detoxifying herb for the treatment of poisoning caused by pesticides, alcohol, heavy metals, and other environmental toxicants. Previous studies have reported that *T. laurifolia* possesses a wide range of pharmacological activities, including antioxidant, anti-inflammatory, and detoxifying effects [1]. Phytochemical investigations have revealed that *T. laurifolia* contains diverse bioactive constituents, particularly phenolic acids, flavonoids, and iridoid glycosides. CA and RA are also widely used as chemical markers for the standardization and quality control of *T. laurifolia* extracts [14,21]. Beyond their role as marker compounds, CA and RA have been extensively reported to exert potent antioxidant, anti-inflammatory, and detoxifying effects by scavenging ROS, inhibiting lipid peroxidation, and preserving endogenous antioxidant defense systems. Furthermore, CA and RA are widely recognized as antioxidant-related bioactive constituents in medicinal plants [22,23,24]. In the present study, both CA and RA were identified as major constituents of the *T. laurifolia* extract.

The DPPH and ABTS assays primarily evaluate the free radical scavenging capacity of antioxidants [25,26], whereas the FRAP assay reflects ferric ion-reducing power and electron-donating capacity [27]. In contrast, the superoxide radical scavenging assay specifically assesses the ability of the extract to eliminate biologically relevant ROS [28,29]. Because each assay represents a distinct antioxidant mechanism, their combined application provides a more comprehensive evaluation of the antioxidant potential of *T. laurifolia* than any single assay alone [30,31,32]. In the present study, the aqueous extract of *T. laurifolia* exhibited significant antioxidant activity in multiple in vitro assays. Consistent with these findings, CA and RA also demonstrated potent antioxidant activities. Their antioxidant capacity is primarily attributed to their hydroxyl-rich structures, which facilitate electron transfer, hydrogen atom donation, and free radical scavenging. These well-documented redox-modulating properties likely contribute substantially to the overall antioxidant activity of the *T. laurifolia* extract [33,34,35,36,37]. Nevertheless, the antioxidant activity of *T. laurifolia* is likely the result of the combined actions of CA, RA, and other phytochemicals present in the extract [38,39].

Organophosphate poisoning is characterized by cholinergic symptoms, including excessive salivation, lacrimation, muscle tremors, respiratory distress, diarrhea, and, in severe cases, convulsions and respiratory failure. These manifestations result from the accumulation of acetylcholine at cholinergic synapses due to inhibition of AChE [40,41]. Because AChE plays an important role in organophosphate toxicity, the effects of *T. laurifolia* extract, CA, and RA on AChE activity were also evaluated. Neostigmine was included to verify the specificity and validity of the experimental system. As expected, it markedly reduced AChE activity, confirming that the method could reliably detect changes in enzyme activity [42]. In contrast, *T. laurifolia* extract, CA, and RA maintained AChE activity under the experimental conditions.

Early apoptosis is a tightly regulated and generally non-inflammatory process. It facilitates the removal of damaged cells before they progress to more harmful forms of cell death, thereby helping to maintain tissue homeostasis [43,44,45]. Accordingly, compounds that promote early apoptosis may facilitate the timely removal of injured cells, thereby helping maintain tissue homeostasis and limiting further cellular damage [46]. The *T. laurifolia* extract induced both early and late apoptosis, with a notable increase in early apoptotic cells at 800 µg/mL, whereas CA and RA predominantly induced late apoptosis. These findings suggest that the apoptotic profile of the extract differs from those of its major phenolic constituents and is characterized, in part, by the induction of early apoptotic responses in LX-2 cells under the experimental conditions. Accordingly, induction of early apoptosis in LX-2 cells may reflect a cellular response associated with the removal of damaged or activated cells, thereby potentially contributing to the maintenance of cellular homeostasis during toxicant-induced injury. Because activated hepatic stellate cells are highly responsive to oxidative stress and play important roles in hepatic injury and tissue remodeling, modulation of apoptosis in these cells may represent a potential biological process associated with the effects of *T. laurifolia* following toxicant exposure [47,48,49,50]. However, the present study did not evaluate hepatic stellate cell apoptosis in vivo, and therefore the relationship between the apoptosis observed in LX-2 cells and hepatoprotective effects in CPF-exposed rats remains to be determined. Collectively, these findings suggest that attenuation of oxidative stress, maintenance of AChE activity, and modulation of apoptosis represent complementary biological activities of *T. laurifolia*. Although the relationship among these activities was not directly investigated, they may collectively contribute to the protective effects of the extract against toxicant-induced cellular injury and provide scientific support for its traditional medicinal use [20,51,52,53,54].

To determine whether these in vitro biological activities translated into detoxifying effects in vivo, the extract was further evaluated in a chlorpyrifos (CPF)-induced toxicity model in Sprague–Dawley rats. CPF, a widely used organophosphate pesticide, is a well-established model for evaluating detoxifying agents due to its ability to induce systemic and hepatic toxicity [17,55,56]. CPF induces excessive ROS production, oxidative stress, mitochondrial dysfunction, and inflammatory responses, leading to cellular injury in multiple organs, particularly the liver [51,57,58].

High doses of CPF (30–50 mg/kg BW) have been reported to induce marked systemic toxicity accompanied by pronounced inflammatory responses and oxidative tissue damage [59,60]. These effects are commonly manifested as body weight loss, altered organ weights, histopathological lesions in target organs, and abnormalities in hematological and clinical biochemical parameters [59,60]. A lower CPF dose of 16 mg/kg BW was selected in the present study to induce oxidative stress while minimizing excessive toxicity [20]. In the present study, CPF administration produced biochemical evidence of toxicity and oxidative stress, but did not result in alterations in body weight, organ weights, or detectable histopathological damage compared with the normal control group. Although some hematological parameters showed statistically significant differences, these changes were relatively small and were not accompanied by pathological abnormalities. Importantly, an increase in ALT activity was observed in the 617 mg/kg group. However, the ALT value in this group (85.50 U/L) remained only mildly elevated compared with the reported reference range for male Sprague–Dawley rats of a comparable age (approximately 36–64 U/L) [61]. In general, clinically relevant hepatocellular injury is typically associated with enzyme elevations exceeding two-fold above the upper reference limit and should be interpreted in conjunction with gross pathological and histopathological findings. In the present study, no abnormalities were observed in gross pathology, histopathological examination, or other liver-related parameters. Therefore, the isolated increase in ALT activity is unlikely to indicate biologically significant hepatic dysfunction and most likely reflects normal biological variability among animals. Collectively, these findings suggest that the CPF dose used in this in vivo study was sufficient to induce oxidative stress without causing detectable organ injury or systemic toxicity. Under these conditions, the antioxidant activity of *T. laurifolia* may have contributed to attenuation of CPF-induced oxidative stress and preservation of endogenous antioxidant defenses.

Because oxidative stress is a major mechanism underlying CPF-induced toxicity, reducing oxidative stress is considered a key pathway through which detoxifying agents exert their protective effects [62,63,64]. CPF-induced toxicity is characterized by increased lipid peroxidation accompanied by depletion of endogenous antioxidant defenses [17,65,66]. Consistent with this mechanism, CPF exposure in the present study significantly increased MDA levels while reducing SOD activity and GSH levels, indicating disruption of cellular redox homeostasis. Pre-treatment with *T. laurifolia* significantly attenuated these alterations, as evidenced by reduced lipid peroxidation together with restoration of SOD activity and GSH levels. These findings suggest that the extract preserved endogenous antioxidant defenses and effectively protected against CPF-induced oxidative stress. Under the present experimental conditions, CPF administration at 16 mg/kg BW in six-week-old rats did not produce marked histopathological changes in liver or kidney tissues. These findings suggest that the current experimental model represents a state of subclinical oxidative stress rather than overt organ injury. Significant alterations in AChE activity, MDA levels, GSH content, and SOD activity nevertheless confirmed the presence of CPF-induced biochemical dysfunction. Therefore, the detoxifying effects observed herein should be interpreted as attenuation of early toxic responses associated with CPF exposure rather than reversal of established tissue damage.

Six-week-old Sprague–Dawley rats were used in the current investigation, whereas our previous study employed four-week-old animals exposed to the same CPF dose (16 mg/kg BW) [20]. Notably, hepatic histopathological alterations were observed in the younger rats but not in the animals evaluated here. Although the experimental conditions were otherwise comparable, differences in animal age may have contributed to the differing hepatic responses. Previous studies have demonstrated that hepatic xenobiotic-metabolizing enzymes, including cytochrome P450 isoforms, undergo substantial maturation during postnatal development, which may influence the metabolism, disposition, and toxicity of xenobiotics in an age-dependent manner. Consequently, age-related differences in xenobiotic metabolism and toxicokinetics may partly explain the absence of histopathological alterations observed in the current experiment [67,68]. Since CPF toxicity is closely associated with hepatic biotransformation processes, age-dependent differences in metabolic capacity may influence the severity of CPF-induced liver injury.

In addition to oxidative stress, CPF exposure significantly reduced AChE activity. This observation is consistent with the established mechanism of organophosphate toxicity, which involves irreversible inhibition of AChE through phosphorylation of its active site [40,66,69]. Interestingly, pre-treatment with *T. laurifolia* significantly restored AChE activity in CPF-exposed rats. The restoration of AChE activity observed in the present study may be associated with the antioxidant effects of the extract. Although the underlying mechanisms were not directly investigated, attenuation of oxidative stress may contribute to the preservation of AChE activity during CPF exposure. Similar protective effects have been reported for *T. laurifolia* and other antioxidant-rich medicinal plant extracts in CPF-induced toxicity models [20,52,70,71].

An integrated interpretation of the present findings may provide further insight into the traditional use of *T. laurifolia* as a detoxifying medicinal plant. Phytochemical analysis identified CA and RA as major phenolic constituents, both of which have been associated with antioxidant and redox-modulating properties [36,72,73,74]. The extract exhibited antioxidant activity in vitro and attenuated CPF-induced oxidative stress in vivo, as evidenced by reduced MDA levels and preservation of endogenous antioxidant defenses, including GSH and SOD. Maintenance of redox homeostasis may help protect cellular components from oxidative damage and contribute to preservation of AChE activity. Together with the observed modulation of apoptosis in LX-2 cells, these findings suggest a coordinated biological response associated with attenuation of CPF-induced oxidative stress and biochemical dysfunction. Although direct causal relationships cannot be established, the convergence of phytochemical, cellular, and animal evidence supports oxidative stress attenuation as a central biological pathway underlying the traditionally recognized detoxifying effects of *T. laurifolia*. It should be noted that the term “detoxification” is used in a specific context in the present study. Here, it refers to attenuation of toxic effects and preservation of endogenous defense mechanisms rather than enhancement of xenobiotic metabolism or elimination.

CPF toxicity is known to involve multiple mechanisms beyond oxidative stress and AChE inhibition, including neurodevelopmental toxicity, endocrine disruption, mitochondrial dysfunction, inflammatory responses, and alterations in intracellular signaling pathways. The present study focused primarily on oxidative stress-related mechanisms and did not investigate other pathways involved in CPF toxicity. In addition, the effects of *T. laurifolia* on CPF metabolism and elimination were not evaluated. Therefore, the findings should be interpreted as evidence of attenuation of CPF-induced oxidative stress and biochemical dysfunction rather than comprehensive protection against all toxic effects of CPF. Accordingly, the present model is more appropriately considered a model of CPF-induced subclinical oxidative stress rather than detectable organ injury. To facilitate interpretation of the experimental doses in a translational context, the administered rat doses were converted to human equivalent doses (HEDs) using body surface area normalization [75]. The doses of 617, 1234, and 2468 mg/kg in rats correspond to approximately 100.05, 200.11, and 400.22 mg/kg in humans, respectively, equivalent to approximately 6.0, 12.0, and 24.0 g/day for a 60-kg adult.

In addition to providing translational context, the present study offers several methodological strengths. A major strength of the present study is the integration of phytochemical characterization, cellular assays, and an in vivo chlorpyrifos exposure model. This multi-level approach allowed evaluation of biological effects from phytochemical composition to cellular responses and whole-animal outcomes, providing a more comprehensive assessment of the detoxifying potential of *T. laurifolia* than studies relying on a single experimental platform.

While these findings provide valuable insight into the detoxifying potential of *T. laurifolia*, several limitations should be acknowledged. First, although the findings suggest that attenuation of oxidative stress contributes to the protective effects of *T. laurifolia*, the underlying molecular mechanisms were not directly investigated. Therefore, the involvement of antioxidant signaling pathways, such as Nrf2-mediated responses, remains to be confirmed. Second, although *T. laurifolia* extract induced apoptosis in LX-2 cells in our in vitro study, this mechanism was not investigated in the present in vivo model. Future studies employing TUNEL staining and caspase-3 immunohistochemistry are warranted to determine whether apoptosis also contributes to the hepatoprotective activity of the extract in vivo. Third, the biological activities reported herein were evaluated using the aqueous extract of *T. laurifolia* rather than its individual constituents. Although CA and RA were identified as major phenolic compounds and may contribute to the observed activities, their specific roles were not directly examined. Furthermore, the contribution of other phytochemicals present in the extract cannot be excluded. Collectively, these limitations highlight the need for further mechanistic and phytochemical investigations to better elucidate the biological basis of the detoxifying effects of *T. laurifolia*.

## 4. Materials and Methods

### 4.1. Plant Material and Extract Preparation

The botanical and taxonomical characteristics of the plant specimen were identified according to the Thai Herbal Pharmacopoeia (THP) 2017 [76]. The plant specimen was identified by Dr. Piyanuch Rojsanga. A voucher specimen (RJ160401) was deposited at the Department of Pharmaceutical Chemistry, Faculty of Pharmacy, Mahidol University, Bangkok, Thailand. Dried leaves of *T. laurifolia* were extracted with distilled water by Ouay Un Osot Co., Ltd., Bangkok, Thailand. Briefly, 50 kg of dried leaf powder were boiled with distilled water at a ratio of 1:10 (*w*/*v*) at 100 °C for 2 h. The extraction process was repeated twice, and the combined extracts were concentrated to one-tenth of the original volume. Maltodextrin was added prior to spray drying using an inlet temperature of 185 °C and an outlet temperature of 86 °C. The dried extract was weighed and calculated as percentage yield (% *w*/*w*).

#### 4.1.1. Chemical Composition Analysis by TLC

Phytochemical characterization of the *T. laurifolia* extract was performed using thin-layer chromatography (TLC). The extract was prepared at a concentration of 10 mg/mL. Standard solutions of CA and RA were prepared by dissolving 1 mg of each compound in methanol in separate 5 mL volumetric flasks. Eight microliters of the extract and each standard solution were applied onto TLC plates as 8 mm bands. TLC analysis was carried out using silica gel 60 F254 as the stationary phase. Two mobile phase systems were used: System I, chloroform: methanol: formic acid (70:30:5), and System II, toluene: ethyl acetate: formic acid (10:9:2). The chromatograms were visualized using NP-PEG reagent under UV light at 366 nm.

#### 4.1.2. Quantitative Analysis of Marker Contents by HPLC

Quantitative analysis of CA and RA in the *T. laurifolia* extract was performed using an HPLC Shimadzu LC-20AC system (Shimadzu Corporation, Kyoto, Japan) equipped with a photodiode array detector and LabSolutions^®^ software. Separation was achieved on an Agilent SB-C18 column (Agilent Technologies, Santa Clara, CA, USA, 5 µm, 3.0 × 150 mm) at 30 °C using gradient elution with 0.02% orthophosphoric acid (mobile phase A) and acetonitrile (mobile phase B). The flow rate was maintained at 1.0 mL/min with a total run time of 40 min and an injection volume of 20 µL.

Standard calibration curves were prepared using caffeic acid (0.3–30 µg/mL) and rosmarinic acid (0.6–60 µg/mL) in methanol. For sample preparation, 20 mg of extract powder were dissolved in distilled water, sonicated for 10 min, adjusted to volume, and filtered through a 0.45 µm PTFE syringe filter prior to analysis. The amounts of CA and RA in the extract were quantified and expressed as mg/g dried extract.

### 4.2. In Vitro Antioxidant Assays

#### 4.2.1. DPPH Testing

The free radical scavenging activity of the extract and isolated compounds was evaluated using the DPPH assay with slight modifications from previously reported methods [77,78,79]. The DPPH reagent was prepared by dissolving DPPH (8 mg) in methanol and adjusting the final volume to 100 mL. Briefly, 20 µL of sample solution was mixed with 180 µL of DPPH reagent in a 96-well plate and incubated in the dark at room temperature for 30 min. Absorbance was measured at 517 nm using a microplate reader (BioTek Instruments, Winooski, VT, USA). Gallic acid and methanol were used as the reference standard and blank control, respectively. The percentage of DPPH radical scavenging activity was calculated using Equation (1):

(1) $$\mathrm{DPPH}\;\mathrm{scavenging}\left(\%\right)=\frac{A_{\mathrm{control}}-A_{\mathrm{sample}}}{A_{\mathrm{control}}}\times 100$$

where $A_{\mathrm{control}}$ is the absorbance of the control reaction and $A_{\mathrm{sample}}$ is the absorbance in the presence of the test sample.

IC_50_ values were calculated by linear regression analysis and expressed as the concentration required to scavenge 50% of DPPH radicals.

#### 4.2.2. ABTS Testing

The ABTS radical scavenging activity was determined according to the method described by Sharopov et al. [80] and Re et al. [25] with slight modifications. ABTS stock solution (7.0 mM) was prepared in deionized water and reacted with potassium persulfate to generate ABTS^+^ radicals. The mixture was incubated in the dark at room temperature for 16 h prior to use. Before analysis, the ABTS^+^ solution was diluted with distilled water to obtain an absorbance of 0.700 ± 0.02 at 630 nm.

For the assay, 10 µL of sample solution was mixed with 190 µL of ABTS working solution in a 96-well plate. After incubation at room temperature for 15 min, absorbance was measured at 630 nm using a microplate reader. Trolox was used as the reference antioxidant. Antioxidant activity was expressed as Trolox equivalent antioxidant capacity (TEAC), calculated from the Trolox calibration curve and normalized to the concentration of the tested extract.

#### 4.2.3. FRAP Testing

The ferric reducing antioxidant power (FRAP) assay was carried out according to previously reported methods [27,81], with slight modifications. This assay evaluates the antioxidant reducing capacity of the samples based on the reduction of the ferric tripyridyltriazine (Fe^3+^–TPTZ) complex to the ferrous form (Fe^2+^–TPTZ), resulting in the formation of a blue-colored complex detectable at 595 nm. The FRAP working reagent was freshly prepared by mixing acetate buffer (300 mM, pH 3.6), TPTZ solution (10 mM in 40 mM HCl), and FeCl_3_·6H_2_O solution (20 mM) at a ratio of 10:1:1. The reagent was pre-incubated at 37 °C before use. Briefly, 10 µL of sample solution at various concentrations was added to a 96-well plate, followed by 190 µL of FRAP reagent. After incubation in the dark at room temperature for 5 min, absorbance was measured at 595 nm using a microplate reader. Ferrous sulfate (FeSO_4_) was used as the reference standard, and appropriate blanks were included for background correction. All measurements were performed in triplicate and expressed as mean ± SEM (n = 3). A standard calibration curve was generated using FeSO_4_, and the antioxidant capacity of the samples was expressed as mM Fe(II)/g dry extract.

#### 4.2.4. Superoxide Testing

Superoxide radical scavenging activity was determined according to previously reported methods [82,83] with slight modifications. Superoxide radicals were generated in a PMS–NADH system and detected by the reduction of nitro blue tetrazolium (NBT). PMS (25 μM), NADH (0.5 mM), and NBT (0.2 mM) were prepared in phosphate buffer (pH 7.4). Briefly, 50 μL each of NBT, NADH, and sample solutions were mixed in a 96-well plate, followed by the addition of 50 μL PMS solution. After incubation at room temperature for 10 min, absorbance was measured at 560 nm using a microplate reader. Gallic acid and phosphate buffer were used as the reference standard and control, respectively. The percentage of superoxide radical scavenging activity and IC_50_ values were calculated using Equation (1).

### 4.3. In Vitro Acetylcholinesterase (AChE) Activity Assay

AChE activity was determined using a modified Ellman colorimetric method [84]. Briefly, test samples dissolved in methanol were incubated with acetylcholinesterase solution (0.22 U/mL, 25 µL) at 37 °C for 15 min. Subsequently, 3 mM 5,5′-dithiobis-(2-nitrobenzoic acid) (DTNB; 125 µL), 15 mM acetylthiocholine iodide (25 µL), and buffer solution (50 µL) were added to the reaction mixture. The mixtures were further incubated at room temperature for 30 min, and absorbance was measured at 412 nm using a microplate reader. Methanol was used as the vehicle control. AChE activity was expressed as a percentage of the vehicle control, which was defined as 100% enzyme activity. The percentage of AChE activity was calculated using Equation (2).

(2) $$\mathrm{AChE}\;\mathrm{activity}(\%)=\frac{A_{\mathrm{sample}}}{A_{\mathrm{control}}}\times 100$$

where $A_{\mathrm{control}}$ is the absorbance of the untreated control reaction, and $A_{\mathrm{sample}}$ is the absorbance of the reaction in the presence of the test sample.

### 4.4. Cell Culture, Cell Proliferation, and Apoptosis Assay

LX-2 human hepatic stellate cells (Merck Millipore, Billerica, MA, USA), an established in vitro model of activated hepatic stellate cells for hepatic fibrosis research, were cultured in DMEM supplemented with 10% FBS and 1% penicillin–streptomycin as previously described by Xu et al. [15]. Cells were maintained at 37 °C in a humidified atmosphere containing 5% CO_2_. The culture medium was replaced every 3 days. Upon reaching 70–80% confluence, cells were harvested and either sub-cultured or used for subsequent experiments.

Cell proliferation was evaluated using a sulforhodamine B (SRB) colorimetric assay as previously described [85]. LX-2 cells were seeded into 96-well plates at a density of 1 × 10^4^ cells/well and incubated at 37 °C in a humidified atmosphere containing 5% CO_2_ for 24 h. The cells were then treated with various concentrations of the extracts and further incubated for 24 h. Cells were fixed with cold trichloroacetic acid (TCA; 10%, *w*/*v*) at 4 °C for 1 h. After washing with tap water and air-drying, the cells were stained with 0.057% SRB solution at room temperature for 30 min. Excess dye was removed using 1% acetic acid, and the plates were allowed to dry. Bound dye was subsequently dissolved in 10 mM Tris base solution, and absorbance was measured at 510 nm [86] using a microplate reader (BioTek Instruments, Winooski, VT, USA).

For annexin V/PI apoptosis testing, LX-2 cells were seeded into 6-well plates at a density of 3 × 10^5^ cells/well and incubated at 37 °C in a humidified atmosphere containing 5% CO_2_ for 24 h. The cells were then treated with various concentrations of the test substances and further incubated for an additional 24 h. Following treatment, the cells were washed twice with cold phosphate-buffered saline (PBS) and detached using trypsin. The collected cells were centrifuged at 1200 rpm for 5 min, washed with cold PBS, and resuspended in binding buffer. Annexin V and propidium iodide (PI) staining was performed using an Annexin V/PI apoptosis detection kit (ImmunoTools, Friesoythe, Germany) according to the manufacturer’s instructions. Briefly, 5 µL each of Annexin V and PI were added to the cell suspension and incubated in the dark for 20 min. After the addition of binding buffer, apoptotic and necrotic cells were analyzed by flow cytometry using a Beckman Coulter CyAn ADP flow cytometer (Beckman Coulter, Brea, CA, USA) [87]. Cells negative for both Annexin V and PI (Annexin V−/PI−) were considered viable cells. Cells positive for Annexin V but negative for PI (Annexin V+/PI−) were considered early apoptotic cells. Cells positive for both Annexin V and PI (Annexin V+/PI+) were considered late apoptotic or secondary necrotic cells, whereas cells negative for Annexin V but positive for PI (Annexin V−/PI+) were considered necrotic cells.

### 4.5. In Vivo Assays

Six-week-old male Sprague–Dawley rats (180–250 g) were obtained from Nomura Siam International Co., Ltd. (Bangkok, Thailand). The animals were housed under controlled environmental conditions (25 ± 1 °C, 60% relative humidity, and a 12 h light/dark cycle) with free access to standard laboratory chow and water. Following a one-week acclimatization period, the animals were used for the experiments. All experimental procedures were approved by the Ethics Committee on the Use of Experimental Animals, Faculty of Medicine, Chiang Mai University, Thailand (Approval No. 21/2562).

#### 4.5.1. Anti-Pesticide Testing

The protective effects of *T. laurifolia* extract against chlorpyrifos (CPF)-induced oxidative stress were evaluated according to the method described previously [55] with slight modifications. Following acclimatization, all rats were weighed and ranked according to body weight. Animals were then allocated to five experimental groups (n = 6 per group) using a weight-matched randomization procedure to minimize selection bias and ensure comparable baseline body weights among groups.

Group 1 served as the normal control and received distilled water.
Group 2 served as the pesticide control and received CPF (16 mg/kg body weight/day).
Groups 3–5 received *T. laurifolia* extract at doses of 617, 1234, and 2468 mg/kg body weight, respectively.

The dose regimen was established based on our preliminary study, which identified 1234 mg/kg body weight as an effective dose of *T. laurifolia* extract. Therefore, a lower dose (617 mg/kg body weight; one-half of the effective dose) and a higher dose (2468 mg/kg body weight; two-fold of the effective dose) were included to evaluate potential dose-dependent effects. The extract was orally administered daily 30 min prior to CPF administration for 16 consecutive days. Body weights were recorded every day throughout the experimental period. At the end of the experimental period, rats were euthanized with thiopental sodium (150 mg/kg, i.p.) before blood and tissue collection. Blood samples were collected by cardiac puncture for each experimental protocol.

#### 4.5.2. Body Weight and Organ Weight Analysis

Body weights were measured on Days 1, 8, and 16 throughout the experimental period to monitor treatment-related changes in growth and general health status. At the end of the experiment, animals were euthanized, and the liver and kidneys were carefully excised, rinsed with ice-cold normal saline to remove residual blood, gently blotted dry with filter paper, and weighed immediately using an analytical balance. Absolute organ weights (g) were recorded for subsequent analysis.

#### 4.5.3. Histopathological Analysis

Representative liver and kidney tissue samples were fixed in 10% neutral buffered formalin, dehydrated through a graded ethanol series, cleared in xylene, and embedded in paraffin. Paraffin-embedded tissues were sectioned at 4 μm thickness and stained with hematoxylin and eosin (H&E). Histopathological alterations were evaluated under a light microscope (Olympus CX-23; Olympus Corporation, Tokyo, Japan). To minimize observer bias, tissue sections were coded prior to evaluation, and the pathologist was blinded to treatment allocation and animal identity throughout the microscopic examination.

#### 4.5.4. Hematological Analysis

Hematological parameters, including red blood cell count (RBC), white blood cell count (WBC), hemoglobin (Hb), hematocrit (Hct), platelet count, mean corpuscular hemoglobin (MCH), mean corpuscular volume (MCV), and mean corpuscular hemoglobin concentration (MCHC), were analyzed using a Mindray BC-5300 Vet automated hematology analyzer (Mindray, Shenzhen, China). Differential white blood cell counts, including neutrophils (Neu), lymphocytes (Lymph), monocytes (Mono), eosinophils (Eos), and basophils (Baso), were also determined.

#### 4.5.5. Clinical Blood Chemistry Analysis

Parameters, including blood urea nitrogen (BUN), creatinine (Cr), aspartate aminotransferase (AST), alanine aminotransferase (ALT), alkaline phosphatase (ALP), direct bilirubin (DB), total bilirubin (TB), albumin (Alb), and total protein (TP), were measured using an automated biochemical analyzer (BX-3010; Sysmex, Kobe, Japan).

#### 4.5.6. Serum Oxidative Stress Biomarkers Analysis

Serum oxidative stress biomarkers, including malondialdehyde (MDA), reduced glutathione (GSH), and superoxide dismutase (SOD), were determined using commercial assay kits according to the manufacturers’ instructions (Elabscience^®^, Houston, TX, USA; Sigma-Aldrich, St. Louis, MO, USA; and Randox Laboratories, Crumlin, UK, respectively).

#### 4.5.7. Acetylcholinesterase (AChE) Activity Analysis

AChE activity was determined using a commercial acetylcholinesterase assay kit based on the hydrolysis of acetylcholine by acetylcholinesterase [84], according to the manufacturer’s instructions (Sigma-Aldrich, St. Louis, MO, USA). Whole blood samples were diluted 1:40 with assay buffer (pH 7.5). Subsequently, 10 µL of each diluted sample was transferred into individual wells of a 96-well plate, followed by the addition of 190 µL of working reagent. The reaction mixtures were gently mixed and incubated at room temperature. Absorbance was measured at 412 nm at 2 and 10 min using a microplate reader. AChE activity was calculated according to Equation (3):

(3) $$AChE\;activity\left(units/L\right)=\frac{A\left(sample\right)\;at\;10\;min-A\left(sample\right)\;at\;2\;min}{A\left(calibrator\right)\;at\;10\;min-A\left(calibrator\right)\;at\;2\;min}\times n\times 200$$

where (A) represents absorbance, 200 is the equivalent activity (units/L) of the calibrator, and (n) is the dilution factor.

### 4.6. Statistical Analysis

All data are presented as mean ± standard error of the mean (SEM). Statistical analyses were conducted using GraphPad Prism version 8 (GraphPad Software, San Diego, CA, USA). Data normality was evaluated using the Shapiro–Wilk test. Parametric data were analyzed by one-way analysis of variance (ANOVA) followed by Tukey’s post hoc multiple comparison test, whereas nonparametric data were analyzed using the Kruskal–Wallis test followed by Dunn’s multiple comparison test. A *p*-value < 0.05 was considered statistically significant.

## 5. Conclusions

This study demonstrates that the aqueous extract of *T. laurifolia* possesses detoxifying activity supported by integrated phytochemical, in vitro, and in vivo evidence. In vitro, the extract exhibited antioxidant activity, maintained acetylcholinesterase activity, and modulated apoptotic responses, predominantly through induction of early apoptosis, in LX-2 hepatic stellate cells. In vivo, the extract attenuated chlorpyrifos-induced subclinical oxidative stress, as evidenced by reduced lipid peroxidation, increased GSH and SOD levels, and recovery of CPF-suppressed AChE activity. Collectively, these findings suggest that attenuation of oxidative stress represents a key mechanism underlying the detoxifying effects traditionally attributed to *T. laurifolia*. The combined phytochemical, cellular, and animal data further indicate that the phenolic-rich extract may help mitigate CPF-induced oxidative stress and associated biochemical dysfunction. These results provide biological support for the traditional use of *T. laurifolia* as a detoxifying medicinal plant. The present study focused primarily on oxidative stress-related mechanisms and did not investigate other toxicological pathways associated with CPF toxicity. Nevertheless, further studies are required to elucidate the underlying molecular mechanisms and other toxicological pathways involved in CPF toxicity.

## Figures and Tables

**Table 2:** Antioxidant activities of *Thunbergia laurifolia* extract and its phenolic compounds as determined by different assays.

Test Substances	DPPH (IC_50_; µg/mL)	Superoxide Radical (IC_50_; µg/mL)	ABTS (TEAC)	FRAP (µM Fe^2+^/µg Extract)
*T. laurifolia*	170.8 ± 5.7	606.7 ± 55.0	0.16 ± 0.02	1.1 ± 0.3
CA	12.4 ± 6.4	85.3 ± 1.5	6.22 ± 0.32	17.4 ± 2.8
RA	12.1 ± 6.8	79.6 ± 5.0	5.60 ± 0.15	20.5 ± 3.3
Gallic acid	2.0 ± 0.1	35.5 ± 1.1	ND	ND

Values are expressed as mean ± SEM (n = 3). TEAC = Trolox equivalent antioxidant capacity. Values were calculated from the Trolox calibration curve and normalized to the concentration of extract. ND = not determined.

**Table 3:** Body weight changes in rats in the chlorpyrifos (CPF)-induced toxicity model.

Experimental Groups	Body Weight (g)		
	Day 1	Day 8	Day 16
Normal control	251.67 ± 4.77	276.67 ± 8.53	296.67 ± 11.67
CPF Control	260.00 ± 6.71	261.67 ± 8.82	270.83 ± 10.52
*T. laurifolia* 617 mg/kg + CPF	267.50 ± 3.10	280.83 ± 6.67	295.17 ± 12.14
*T. laurifolia* 1234 mg/kg + CPF	269.17 ± 8.41	293.33 ± 10.93	324.17 ± 15.30
*T. laurifolia* 2468 mg/kg + CPF	263.50 ± 2.93	288.33 ± 8.63	312.50 ± 10.14

Values are expressed as mean ± SEM (n = 6).

**Table 4:** Liver and kidney weights of rats in the chlorpyrifos (CPF)-induced toxicity model.

Experimental Groups	Liver Weight (g)	Kidney Weight (g)
Normal control	8.19 ± 0.49	1.06 ± 0.04
CPF control	9.26 ± 0.44	1.21 ± 0.05
*T. laurifolia* 617 mg/kg + CPF	9.72 ± 0.66	1.13 ± 0.06
*T. laurifolia* 1234 mg/kg + CPF	9.34 ± 0.76	1.22 ± 0.08
*T. laurifolia* 2468 mg/kg + CPF	10.05 ± 0.80	1.26 ± 0.08 *

Values are expressed as mean ± SEM (n = 6). * *p* < 0.05 compared with the CPF control group.

**Table 5:** Hematological parameters in the chlorpyrifos (CPF)-induced toxicity model.

Parameters	Normal Control	CPF Control	*T. laurifolia* Extract + CPF		
			617 mg/kg	1234 mg/kg	2468 mg/kg
RBC (×10^6^/µL)	7.31 ± 0.10	8.09 ± 0.29 ^a^	7.69 ± 0.19	7.53 ± 0.17	7.57 ± 0.23
Hb (g/dL)	13.30 ± 0.11	14.75 ± 0.41	13.85 ± 0.38	13.70 ± 0.26 *	13.65 ± 0.38 *
Hct (%)	40.33 ± 0.32	44.98 ± 1.31 ^a^	42.07 ± 1.07	42.23 ± 1.03	42.72 ± 1.50
MCV (fL)	55.22 ± 0.53	55.70 ± 0.74	54.78 ± 0.99	56.12 ± 0.35	56.37 ± 0.68
MCH (pg)	18.20 ± 0.21	18.27 ± 0.20	18.00 ± 0.32	18.20 ± 0.14	18.03 ± 0.20
MCHC (g/dL)	32.97 ± 0.11	32.80 ± 0.26	32.90 ± 0.20	32.43 ± 0.23	32.00 ± 0.27 *
PLT (×10 ^5^/µL)	9.38 ± 0.47	8.85 ± 0.26	8.65 ± 0.31	8.48 ± 0.26	8.41 ± 0.34

Values are expressed as mean ± SEM (n = 6). ^a^ *p* < 0.05 compared with the normal control group. * *p* < 0.05 compared with the CPF control group. Abbreviations: RBC, red blood cell; Hb, hemoglobin; Hct, hematocrit; MCV, mean corpuscular volume; MCH, mean corpuscular hemoglobin; MCHC, mean corpuscular hemoglobin concentration; PLT, platelet count.

**Table 6:** Differential white blood cell counts in the chlorpyrifos (CPF)-induced toxicity model.

Parameters (×10^3^ Cells/µL)	Normal Control	CPF Control	*T. laurifolia* Extract + CPF		
			617 mg/kg	1234 mg/kg	2468 mg/kg
WBC	5.60 ± 0.42	7.10 ± 0.91	6.04 ± 0.94	5.92 ± 0.64	6.06 ± 0.99
Neu	1.45 ± 0.11	2.14 ± 0.47	1.39 ± 0.17	1.64 ± 0.23	1.69 ± 0.36
Lym	3.73 ± 0.41	3.79 ± 0.53	4.17 ± 0.84	3.83 ± 0.59	3.72 ± 0.55
Mono	0.35 ± 0.07	1.08 ± 0.32 ^a^	0.41 ± 0.05 *	0.38 ± 0.11 *	0.55 ± 0.14 *
Eos	0.08 ± 0.01	0.09 ± 0.01	0.08 ± 0.02	0.07 ± 0.01	0.10 ± 0.02
Baso	0.00 ± 0.00	0.00 ± 0.00	0.00 ± 0.00	0.00 ± 0.00	0.00 ± 0.00

Values are expressed as mean ± SEM (n = 6). ^a^ *p* < 0.05 compared with the normal control group. * *p* < 0.05 compared with the CPF control group. Abbreviations: WBC, white blood cell; Neu, neutrophil; Lym, lymphocyte; Mono, monocyte; Eos, eosinophil; Baso, basophil.

**Table 7:** Serum chemical parameters in the chlorpyrifos (CPF)-induced toxicity model.

Parameters	Normal Control	CPF Control	*T. laurifolia* Extract + CPF		
			617 mg/kg	1234 mg/kg	2468 mg/kg
BUN (mg/dL)	9.85 ± 0.67	12.72 ± 0.82	12.33 ± 1.78	9.65 ± 0.84	12.85 ± 1.55
Cr (mg/dL)	0.61 ± 0.02	0.70 ± 0.04 ^a^	0.62 ± 0.02 *	0.60 ± 0.02 *	0.64 ± 0.02
TP (g/dL)	5.43 ± 0.08	5.65 ± 0.15	5.50 ± 0.22	5.08 ± 0.06 *	5.28 ± 0.15
Alb (g/dL)	2.83 ± 0.04	3.08 ± 0.05	2.93 ± 0.06	2.85 ± 0.04	2.83 ± 0.05
TB (mg/dL)	0.09 ± 0.01	0.09 ± 0.02	0.09 ± 0.02	0.13 ± 0.04	0.09 ± 0.01
DB (mg/dL)	0.06 ± 0.00	0.06 ± 0.00	0.06 ± 0.00	0.05 ± 0.00	0.06 ± 0.00
AST (U/L)	94.83 ± 3.05	144.33 ± 17.58	155.00 ± 41.70	92.17 ± 1.40	98.33 ± 9.08
ALT (U/L)	23.33 ± 1.43	32.00 ± 3.44	85.50 ± 40.12 *	28.50 ± 1.61	24.50 ± 1.89
ALP (U/L)	92.67 ± 2.87	82.50 ± 3.68	71.33 ± 6.00	76.17 ± 3.76	75.67 ± 6.94

Values are expressed as mean ± SEM (n = 6). ^a^ Significantly different from the normal rats (distilled water), *p* < 0.05. * Significantly different from the control group (distilled water + chlorpyrifos), *p* < 0.05. Abbreviations: BUN, blood urea nitrogen; Cr, creatinine; TP, total protein; Alb, albumin; TB, total bilirubin; DB, direct bilirubin; AST, aspartate aminotransferase; ALT, alanine aminotransferase; ALP, alkaline phosphatase.

**Table 8:** Oxidative stress biomarkers in the chlorpyrifos (CPF)-induced toxicity.

Experimental Groups	MDA (nmol/mg Protein)	GSH (nmol/mL)	SOD (U/mg Protein)
Normal control	0.083 ± 0.004	35.1 ± 8.6	0.038 ± 0.002
CPF control	0.111 ± 0.004 **	14.0 ± 0.7 **	0.027 ± 0.005 *
*T. laurifolia* 617 mg/kg + CPF	0.098 ± 0.004 #	22.1 ± 1.4 #	0.036 ± 0.002 #
*T. laurifolia* 1234 mg/kg + CPF	0.098 ± 0.005 #	25.3 ± 2.9 #	0.038 ± 0.003 #
*T. laurifolia* 2468 mg/kg + CPF	0.090 ± 0.004 ##	27.8 ± 1.7 #	0.037 ± 0.002 #

Data are presented as mean ± SEM (n = 6). * *p* < 0.05, ** *p* < 0.01 compared with the normal control group (distilled water). # *p* < 0.05 and ## *p* < 0.01 compared with the CPF-treated group.

## Data Availability

The original contributions presented in this study are included in the article. Further inquiries can be directed to the corresponding author.
